# Clinical features and FLAIR radiomics nomogram for predicting functional outcomes after thrombolysis in ischaemic stroke

**DOI:** 10.3389/fnins.2023.1063391

**Published:** 2023-02-22

**Authors:** Qingqing Xu, Yan Zhu, Xi Zhang, Dan Kong, Shaofeng Duan, Lili Guo, Xindao Yin, Liang Jiang, Zaiyi Liu, Wanqun Yang

**Affiliations:** ^1^Department of Radiology, The Affiliated Huaian No. 1 People’s Hospital of Nanjing Medical University, Huaian, China; ^2^GE HealthCare, Shanghai, China; ^3^Department of Radiology, Nanjing Medical University Affiliated Nanjing Hospital, Nanjing, China; ^4^Department of Radiology, Guangdong Academy of Medical Sciences, Guangdong Provincial People’s Hospital, Guangzhou, China

**Keywords:** acute ischaemic stroke, diffusion-weighted imaging, outcome, radiomics, nomogram

## Abstract

**Objective:**

We explored whether radiomics features extracted from diffusion-weighted imaging (DWI) and fluid-attenuated inversion recovery (FLAIR) images can predict the clinical outcome of patients with acute ischaemic stroke. This study was conducted to investigate and validate a radiomics nomogram for predicting acute ischaemic stroke prognosis.

**Methods:**

A total of 257 patients with acute ischaemic stroke from three clinical centres were retrospectively assessed from February 2019 to July 2022. According to the modified Rankin scale (mRS) at 3 months, the patients were divided into a favourable outcome group (mRS of 0–2) and an unfavourable outcome group (mRS of 3−6). The high-throughput features from the regions of interest (ROIs) within the radiologist-drawn contour by AK software were extracted. We used two feature selection methods, minimum redundancy and maximum (mRMR) and the least absolute shrinkage and selection operator algorithm (LASSO), to select the features. Three radiomics models (DWI, FLAIR, and DWI-FLAIR) were established. A radiomics nomogram with patient characteristics and radiomics signature was built using a multivariate logistic regression model. The performance of the nomogram was evaluated in the test and validation sets. Ultimately, decision curve analysis was implemented to assess the clinical value of the nomogram.

**Results:**

The FLAIR, DWI, and DWI-FLAIR radiomics model exhibited good prediction performance, with area under the curve (AUCs) of 0.922 (95% CI: 0.876−0.968), 0.875 (95% CI: 0.815−0.935), and 0.895 (95% CI: 0.840−0.950). The radiomics nomogram with clinical characteristics including the overall cerebral small vessel disease (CSVD) burden score, hemorrhagic transformation (HT) and admission National Institutes of Health Stroke Scale score (NIHSS) score and the FLAIR Radscore presented good discriminatory potential in the training set (AUC = 0.94; 95% CI: 0.90−0.98) and test set (AUC = 0.94; 95% CI: 0.87−1), which was validated in the validation set 1 (AUC = 0.95; 95% CI: 0.88−1) and validation set 2 (AUC = 0.90; 95% CI: 0.768−1). In addition, it demonstrated good calibration, and decision curve analysis confirmed the clinical value of this nomogram.

**Conclusion:**

This non-invasive clinical-FLIAR radiomics nomogram shows good performance in predicting ischaemic stroke prognosis after thrombolysis.

## 1. Introduction

Stroke is the second leading cause of death and disability worldwide and the leading cause of death in China (GBD 2019 Stroke Collaborators., 2021). Ischaemic stroke (IS) accounts for 60−80% of all strokes and places a great burden on society due to its high mortality and disability rate (Cao et al., 2021). Recombinant tissue plasminogen activator alteplase (RT-PA) is the most effective drug for the treatment of acute ischaemic stroke. Although the majority of patients experience remission within the next 24 to 72 h, a significant number of patients continue to have dysfunction after thrombolytic therapy. Acute ischaemic stroke has a certain probability of hemorrhagic transformation (HT). Patients receiving intravenous thrombolytic therapy have an increased risk of hemorrhagic transformation, which may reduce or offset the benefit of thrombolytic therapy. Therefore, it is necessary to evaluate the basic clinical data of patients after they enter the emergency department to ensure the safety and effectiveness of intravenous thrombolysis. Cerebral small vessel disease (CSVD) refers to a series of pathological, imaging and clinical syndromes caused by various causes of cerebral arterioles, arterioles, venules, and capillaries (Wardlaw et al., 2013). In China, AIS caused by CSVD accounts for 25−50% (Huang, 2015). Most CSVD patients have an insidious onset and varied clinical manifestations. The presence and severity of symptoms depend on the location, degree and number of lesions. MRI findings can be used as a means to identify CSVD, mainly including residual small subcortical infarct (RSSI), cerebral microbleeds (CMB), white matter hyperintensities (WMH), perivascular spaces (PVS) and brain atrophy. This study retrospectively included the general clinical data and MRI findings of CSVD in patients with acute cerebral infarction undergoing intravenous thrombolysis who were followed up for 90 days. The purpose of this study was to analyse the correlation between the overall CSVD burden and the long-term prognosis of acute ischaemic stroke patients undergoing intravenous thrombolysis. Radiomics is a novel developed data analysis technique that can transform medical images into high-throughput quantitative features, assess the heterogeneity of diseased tissue, and reflect the physiological and pathological status and has been applied to the prediction of clinical outcomes. At present, radiomics has a promising application prospect in stroke, including the diagnosis of stroke (Peter et al., 2017),early prediction of clinical outcome (Wen et al., 2020) and evaluation of medium and long term prognosis (Tang et al., 2020; Quan et al., 2021; Wang et al., 2021; Zhou et al., 2022). Wen et al. (2020) developed a model based on radiological features extracted from computed tomography non-contrast computed tomography (NCCT) and computed tomography angiography (CTA) to predict the development of malignant acute middle cerebral Artery Infarction (mMCAi) in patients with cerebral infarction. Several recent studies have shown that the clinical-radiomics model extracted from diffusion-weighted imaging (DWI), fluid attenuated inversion recovery (FLAIR) or apparent diffusion coefficient (ADC) achieved satisfactory performance in predicting AIS outcomes (Tang et al., 2020; Quan et al., 2021; Wang et al., 2021; Zhou et al., 2022). Most patients with ischaemic stroke receive only routine sequences, including DWI, ADC and FLAIR. DWI can accurately describe the tissue spread and reflect the microstructure of the lesions, and the FLAIR sequence has a higher resolution and can provide more useful information. However, ADC represents the mean value of infarcts. Currently, no studies to date have combined radiomics and the CSVD burden score to predict the prognosis of AIS patients. Therefore, the purpose of this study was to explore the predictive value of DWI and FLAIR-based radiomics combined with the CSVD burden score in ischaemic stroke and to create a method that can be used in the management strategy of ischaemic stroke. Finally, an independent external validation set was used to validate the performance of the radiomics nomogram.

## 2. Subjects and methods

### 2.1. Subjects

**FIGURE 1:**
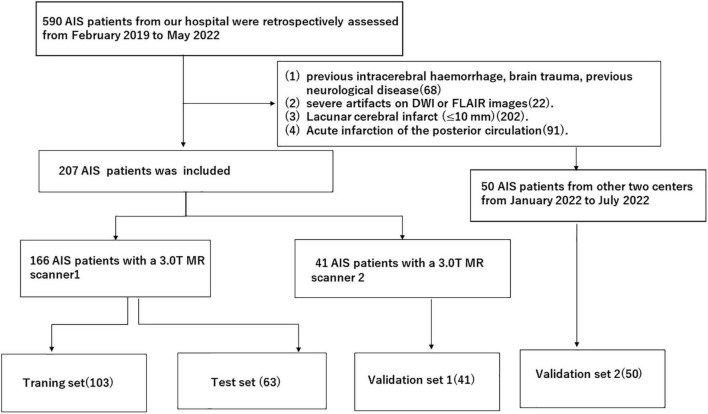
Distribution diagram of enrolled subjects in training set, test set and external validation sets.

A total of 257 patients from three clinical centres with acute infarction diagnosed by DWI after thrombolysis from February 2019 to July 2022 were retrospectively enrolled. Then, 166 patients from our hospital with a Siemens MR scanner were assigned to the training and test sets, and 41 patients from our hospital with a Philips MR scanner were assigned to the external validation set1 and 50 patients from other two clinical centres were assigned to external validation set 2. Distribution diagram of enrolled subjects in training set, test set, and external validation sets was showed in Figure 1. The inclusion criteria were as follows: (1) the time interval from onset to MR examination was ≤72 h; and (2) the patient had no old cerebral infarction and could live independently before the infarction. The exclusion criteria were as follows: (1) previous intracerebral haemorrhage, brain trauma, previous neurological disease, and severe artefacts on DWI or FLAIR images. (2) Lacunar cerebral infarct (≤ 10 mm). (3) Acute infarction of the posterior circulation. This study was a retrospective study and was approved by the ethics committee of our hospital. The requirement for informed consent was waived. Authors confirm that all methods were carried out in accordance with institutional guidelines and regulations.

### 2.2. Methods

#### 2.2.1. DWI and FLAIR examination

A total of 166 acute ischaemic stroke patients underwent MRI examination with a 3.0T MR scanner (Siemens, Verio, Germany). The MRI protocol included axial FLAIR and DWI. The imaging protocol parameters were as follows: FLAIR [TR 9000 ms, TE 100 ms, inversion time (TI) 2500 ms], visual field (FOV) 220 × 220, matrix 256 × 256, DWI (TR 6700 ms, TE 100 ms), FOV 220 × 220, matrix 192 × 192, B value 1000 s/mm^2^. Forty-one patients underwent MRI examination with a 3.0T MR scanner (Philips, Ingenia 3.0cx, Netherlands). The MRI protocol included the imaging protocol parameters were as follows: FLAIR (TR 9000 ms, TE 137 ms, TI 2500 ms), visual field (FOV) 230 × 186 × 125, matrix 328 × 174 × 21, DWI (TR 2585 ms, TE 81 ms), FOV 220 × 220 × 125, matrix 136 × 110 × 21, B value 1000 s/mm^2^. Thirty-six patients underwent MRI examination with a 3.0T MR scanner (Philips, Achieva Tx3.0, Netherlands). The imaging protocol parameters were as follows: FLAIR (TR 11000 ms, TE 125 ms, TI 2800 ms), visual field (FOV) 210 × 210 × 118, matrix 140 × 109, DWI (TR 2245 ms, TE 90 ms), FOV 220 × 220 × 125, matrix 136 × 110 × 21, B value 1000 s/mm^2^. Fourteen patients underwent MRI examination with a 3.0T MR scanner (Philips, Ingenia 3.0cx, Netherlands). The MRI protocol included FLAIR axial, DWI axial scans. The imaging protocol parameters were as follows: FLAIR (TR 9000 ms, TE 120 ms, TI 2600 ms), visual field (FOV) 230 × 230, matrix 356 × 151, DWI (TR 2501 ms, TE 98 ms), FOV 230 × 230, matrix 152 × 122, B value 1000 s/mm^2^.

#### 2.2.2. Clinical data

The demographic and clinical data included sex, age, systolic blood pressure (SP), diastolic blood pressure (DP), MRI CSVD burden score, HT, baseline NIHSS score on admission and mRS score at 90 days. The MRI CSVD burden score was performed according to the scale established by Staals, with a total score of 0–4, and the brain damage caused by CSVD was greater if the score was higher (Staals et al., 2014). One point was recorded for each of the following: (1) any lacune; (2) periventricular WMHs (Fazekas score 2 or 3); (3) any CMB; and (4) moderate to severe (grade 2–4) PVS in the basal ganglia. We calculated an overall CSVD burden score ranging from 0 to 4 with the above 4 imaging markers, and all the scores were assessed by 2 experienced neuroradiologists. The Fazekas scale was used to evaluate the sum of perivascular WMHs and deep WMHs, and the score ranged from 0 to 6. The PVS score was selected from the basal ganglia and central semioval region, and a 4-point scale was used to grade the severity of PVS (Wenli et al., 2021).

#### 2.2.3. Image segmentation and data analysis

##### 2.2.3.1. ROI segmentation and high-throughput feature extraction

Two experienced radiologists (Dr. A, Dr. B) manually segmented lesions on DWI using ITK-SNAP^1^ software and then duplicated the region of interest (ROI) on DWI to the corresponding FLAIR sequence. Fifty patients were randomly selected and segmented by doctors with 5 years and 10 years of experience in neuroimaging diagnosis. The doctors with 5 years of experience segmented the data twice with an interval of 2 weeks between segmentations. A physician with 10 years of experience segmented the data once. The correlation coefficient (ICC) was used to test the intraobserver and intergroup ROI consistency (ICC > 0.75 indicates good agreement). High-throughput features were extracted according to the ROI of each case, including first-order features, shape features, and texture features.

##### 2.2.3.2. Establishment and evaluation of the models

In this study, a feature variable dataset was composed of 166 cases segmented by physicians with 5 years of experience, and the dataset was randomly divided into a training set and a test set at a ratio of 6:4. The data of the training set were used for feature screening and constructing the prediction model, and the data of the test set were used to verify the effect of the model internally. The area under the curve (AUC), sensitivity, specificity and accuracy of the receiver operating characteristic (ROC) curve were used to evaluate the validity and reliability of the prediction model for acute ischaemic stroke after thrombolysis. Features with good repeatability and stability were used to establish the Radscore. The Radscore was calculated by linear fusion of selected features. The calibration curve was used to evaluate the prediction effect of the model. The decision curve was used to evaluate the potential clinical net benefit of the prediction model.

#### 2.2.4. Statistical methods

SPSS 20.0 was used for statistical analysis, the independent sample *t* test was used for the ages of patients in the two groups, and the χ2 test was used for sex distribution differences. A *P* value < 0.05 was considered as a statistically significant difference. All statistical analyses were performed with R software, version 3.4.0. The “DescTools” package was used for ICC calculation, and the “Caret” package was used for data grouping, Spearman correlation analysis and calibration analysis. The “glmnet” package was used for LASSO regression analysis and to construct the Radscore. The “pROC” package was used to plot ROC curves and calculate the characteristics and sensitivity of the models.

## 3. Results

### 3.1. General patient information

**FIGURE 2:**
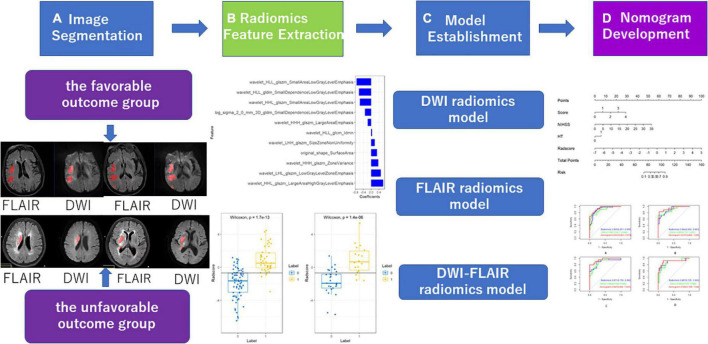
Workflow of radiomics analysis of the experiment.

The study flowchart included image segmentation, feature extraction, model establishment, and nomogram (Figure 2). A total of 166 patients from our hospital with acute ischaemic stroke were divided into two groups. The favourable outcome group comprised 103 cases (62 males and 41 females) and 63 cases (42 males and 21 females) in the unfavourable outcome group. The clinical data (the overall CSVD burden score, HT, age, sex, SP, DP, admission NIHSS score) and imaging characteristics of the training set and the test set were analysed by the chi-square test, and logistic regression analysis was performed with *P* < 0.1 as the inclusion criterion. In the training set, the overall CSVD burden score, HT, admission NIHSS score and Radscore were statistically significant (Table 1), while in the test set, the NIHSS score and Radscore were significantly different (*P* < 0.1), and there was no significant difference in outcome distribution between the two sets (Table 2).

**TABLE 1:** Patient features in the training sets and test sets.

Variable	The training sets				The test sets		
	Level	The favourable outcome group (*n* = 73)	The unfavourable outcome group (*n* = 45)	*P*-value	The favourable outcome group (*n* = 30)	The unfavourable outcome group (*n* = 18)	*P*-value
The overall CSVD burden score	0	5 (6.8)	0 (0)	0.068	3 (10)	0 (0)	
	1	31 (42.5)	12 (26.7)		10 (33.3)	3 (16.7)	
	2	28 (38.4)	23 (51.1)		13 (43.3)	9 (50)	
	3	9 (12.3)	9 (20.0)		4 (13.3)	5 (27.8)	
	4	0 (0.0)	1 (2.2)		0 (0)	1 (5.6)	0.200
HT	No	67 (91.8)	27 (60.0)	<1^e^^−04^	27 (90)	13 (72.2)	
	Yes	6 (8.2)	18 (40.0)		3 (10)	5 (27.8)	0.230
Sex	Female	27 (37.0)	13 (28.9)		14 (46.7)	8 (44.4)	
	Male	46 (63.0)	32 (71.1)	0.482	16 (53.3)	10 (55.6)	1.00
Hypertension	No	21 (28.8)	14 (31.1)	0.787	6 (20)	5 (27.8)	0.535
	Yes	52 (71.2)	31 (68.9)		24 (80)	13 (72.2)	
Diabetes	No	56 (76.7)	35 (77.8)	0.894	23 (76.7)	13 (16.2)	0.731
	Yes	17 (23.3)	10 (22.2)		7 (23.3)	5 (83.8)	
Hyperlipemia	No	62 (84.9)	41 (91.1)	0.487	28 (93.3)	17 (94.4)	1.000
	Yes	11 (15.1)	4 (8.9)		2 (6.7)	1 (5.6)	
Coronary heart disease	No	67 (91.8)	41 (91.1)	1.000	27 (90)	17 (94.4)	1.000
	Yes	6 (8.2)	4 (8.9)		3 (10)	1 (5.6)	
Atrial fibrillation	No	56 (76.7)	31 (68.9)	0.348	26 (86.7)	11 (61.1)	0.092
	Yes	17 (23.3)	14 (31.1)		4 (13.3)	7 (38.9)	
Smoking	No	59 (80.8)	36 (80)	0.913	24 (80)	15 (83.3)	1.000
	Yes	14 (19.2)	9 (20)		6 (20)	3 (16.7)	
Drink	No	68 (89)	42 (93.3)	1.000	30 (100)	17 (94.4)	0.375
	Yes	5 (11)	3 (6.7)		0 (0)	1 (5.6)	
Age, mean (SD)		64.6 (12.6)	66.1 (11.3)	0.529	66.7 (13)	67.5 (14.5)	0.843
DP, mean (SD)		148 (21.6)	147.2 (15.4)	0.847	150 (20.2)	155.1 (19.2)	0.391
SP, mean (SD)		85.2 (13.4)	87 (11.1)	0.431	84 (13.4)	87.4 (12.7)	0.382
Admission NIHSS, mean (SD)		8.1 (3.7)	15.8 (6)	<1^e^^−04^	8.2 (3.4)	17.3 (6.4)	<1e-04
Radscore, median (IQR)		−1.6 (−3.0, −0.7)	0.5 (−0.1, 1.7)	<1^e–04^	−1.9 (−2.6, −0.8)	−0.7 (−3.0, 2.0)	<1e−04

DP, diastolic blood pressure; SP, systolic pressure; IQR, interquartile range; NIHSS, National Institutes of Health Stroke Scale; HT, hemorrhagic transformation.

**TABLE 2:** Population characteristics of patients with AIS in the training and test sets.

Variable	Level	Training (*n* = 118)	Test (*n* = 48)	*P*-value
The overall CSVD burden score	0	5 (4.2)	3 (6.2)	0.752
	1	43 (36.4)	13 (27.1)	
	2	51 (43.2)	22 (45.8)	
	3	18 (15.3)	9 (18.8)	
	4	1 (0.8)	1 (2.1)	
HT	No	94 (79.7)	40 (83.3)	0.744
	Yes	24 (20.3)	8 (16.7)	
Sex	Female	40 (33.9)	22 (45.8)	0.206
	Male	78 (66.1)	26 (54.2)	
Hypertension	No	35 (29.7)	11 (22.9)	0.379
	Yes	83 (70.3)	37 (77.1)	
Diabetes	No	91 (77.1)	36 (75)	0.770
	Yes	27 (22.9)	12 (25)	
Hyperlipemia	No	103 (87.2)	45 (93.8)	0.348
	Yes	15 (12.8)	3 (6.2)	
Coronary heart disease	No	108 (91.5)	44 (91.7)	1.000
	Yes	10 (8.5)	4 (8.3)	
Atrial fibrillation	No	87 (73.7)	37 (77.1)	0.652
	Yes	31 (26.3)	11 (22.9)	
Smoking	No	95 (80.5)	39 (81.2)	0.913
	Yes	23 (19.5)	9 (18.8)	
Drink	No	110 (93.2)	47 (97.9)	0.405
	Yes	8 (6.8)	1 (2.1)	
Age, mean (SD)		65.2 (12.1)	67 (13.4)	0.393
DP, mean (SD)		147.7 (19.4)	151.9 (19.8)	0.203
SP, mean (SD)		85.9 (12.6)	85.2 (13.1)	0.772
Admission NIHSS, mean (SD)		11 (6)	11.6 (6.4)	0.592
Radscore, median (IQR)		−0.7 (−2.2, 0.4)	−0.9 (−2.2, 0.3)	0.842

DP, diastolic blood pressure; SP, systolic pressure; IQR: interquartile range; NIHSS, National Institutes of Health Stroke Scale; HT, hemorrhagic transformation.

### 3.2. Extraction and selection of radiomic features and establishment of radiomic signatures

**FIGURE 3:**
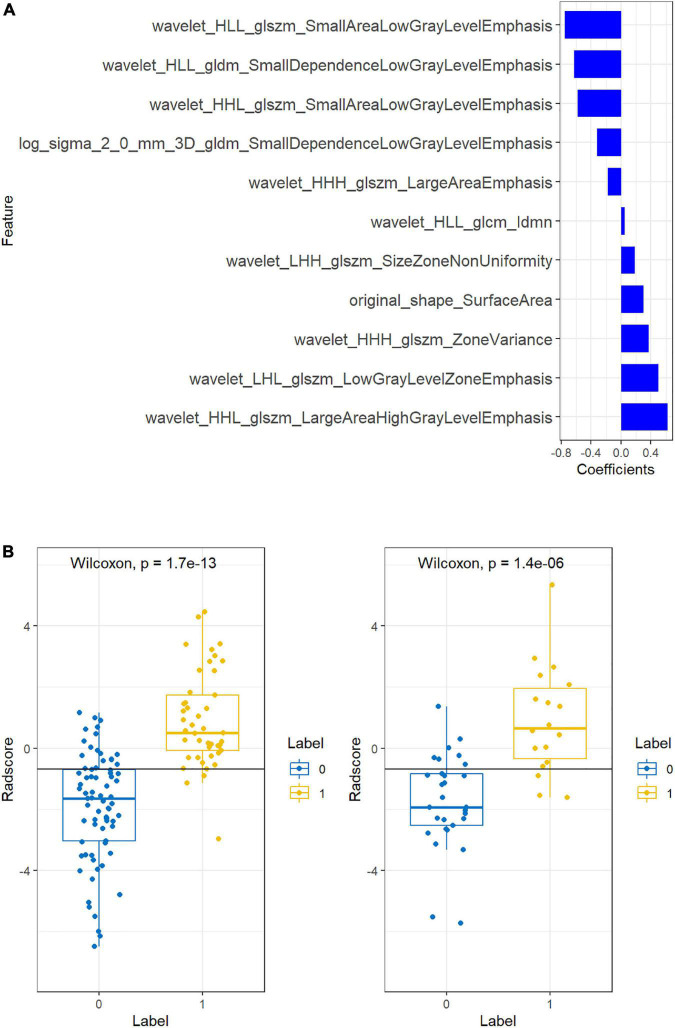
Feature coefficients **(A)** and radiomics scores **(B)**.

The intraobserver and interobserver ICC values were 0.89 and 0.81, respectively, suggesting good consistency. The segmentation dataset of all images for subsequent feature extraction and screening and the model establishment was composed of the data after the first segmentation by the doctor with 5 years of experience. The AUC value of the FLAIR radiomics model in evaluating the prognosis of thrombolysis in acute ischaemic stroke was 0.922 (95% CI: 0.876–0.968), which was higher than that of the DWI radiomics model (AUC = 0.875, 95% CI: 0.815–0.935) and DWI-FLAIR radiomics model (AUC = 0.895, 95% CI: 0.840–0.950), although there was no significant difference between the three models. Based on the FLAIR radiomics model, we first performed mRMR to remove redundant and irrelevant features and retained 30 features and then performed LASSO dimensionality reduction processing to finally screen 11 features with non-zero coefficients, including 1 shape feature and 10 texture features. The parameters and coefficients of each feature are shown in Figure 3A. Subsequently, the radiomics label radiomics score (RAD-score) (Figure 3B) was established to reflect the distribution of the good prognosis group and poor prognosis group in the training group and the test group.

Figure 3A shows the feature parameters of the FLAIR radiomics model. Figure 3B shows the Radscore of the FLAIR radiomics model for the training group and test group. The blue bar below baseline 0 indicates patients with poor prognosis prediction, while the yellow bar above baseline 0 indicates patients with good prognosis prediction. The cross part represents the model prediction error, and the overall prediction effect is good.

### 3.3. Development of the radiomics nomogram

**FIGURE 4:**
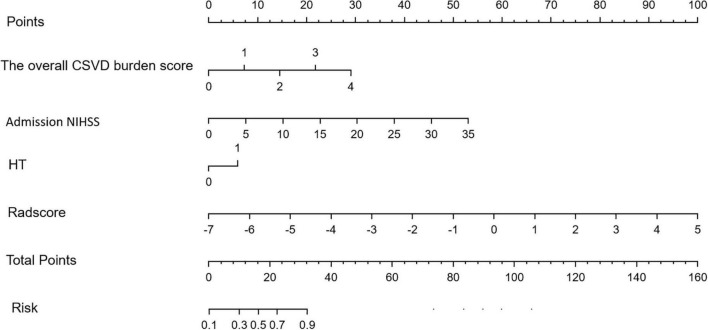
Radiomics nomogram for predicting the clinical functional outcome of ischaemic stroke.

**FIGURE 5:**
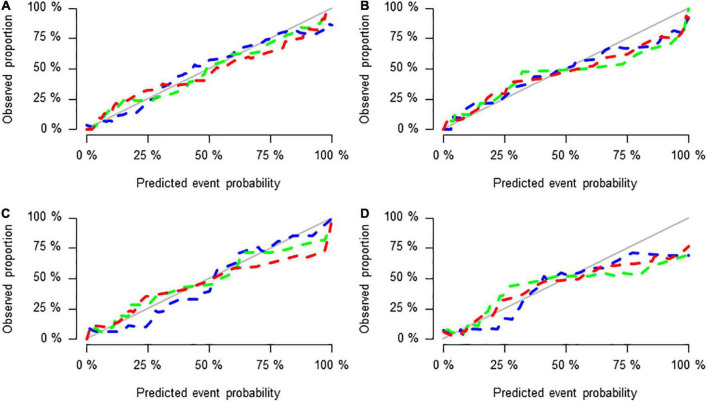
Calibration curves of the three groups of models in the training set **(A)**, test set **(B)**, validation set 1 **(C)**, and validation set 2 **(D)**.

**FIGURE 6:**
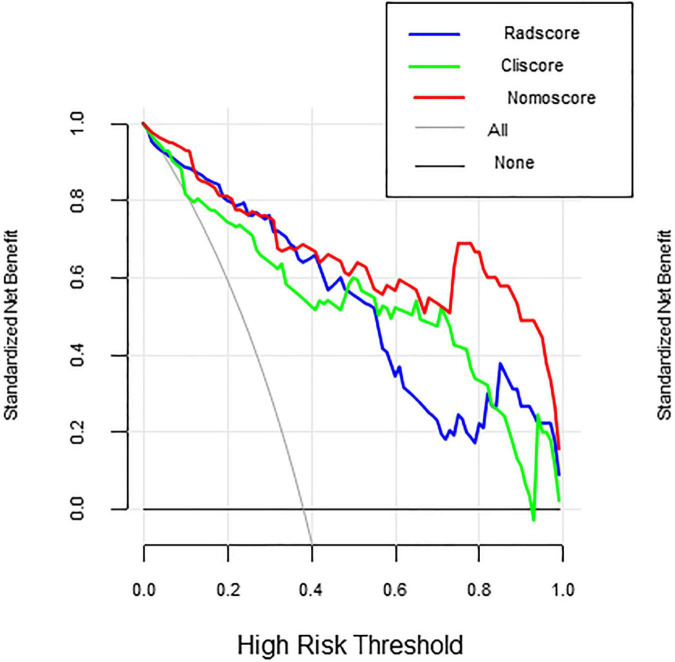
Decision curves.

**FIGURE 7:**
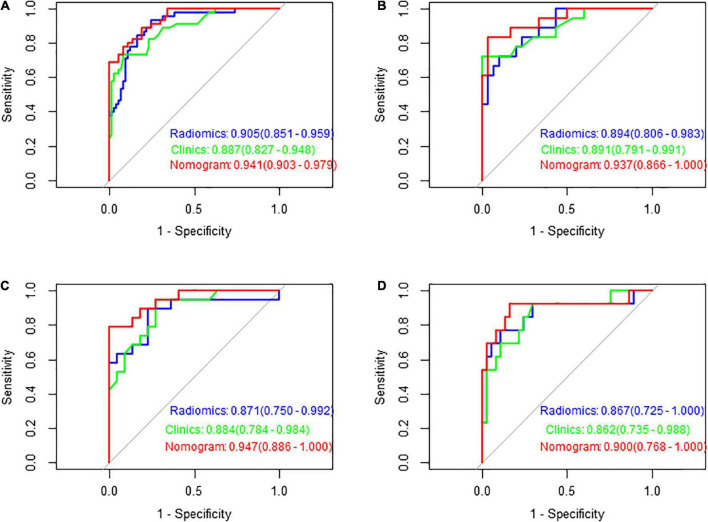
Receiver operating characteristic (ROC) curves of the three models in predicting the clinical functional outcomes of ischaemic stroke in the training set **(A)**, test set **(B)**, validation set 1 **(C)**, and validation set 2 **(D)**.

Multivariate logistic regression analysis showed that the overall CSVD burden score (OR = 1.96, 95% CI: 0.99∼3.86), HT (OR = 1.37, 95% CI: 1.21∼1.56) and NIHSS score (OR = 2.87, 95% CI: 0.77∼10.72) were independent predictors of prognosis in patients with acute ischaemic stroke after thrombolysis (Table 3). Finally, the overall CSVD burden score (OR = 2.7, 95% CI: 1.11∼6.60), HT (OR = 2.24, 95% CI: 0.45∼11.01), NIHSS score (OR = 1.23, 95% CI: 1.05∼1.44) and Radscore (OR = 3.13, 95% CI: 1.73∼5.67) were selected in the training set to create a radiomics nomogram (Table 4 and Figure 4). The prognosis of the training and test sets after thrombolytic therapy for predicting AIS was evaluated by calibration curves (Figure 5). Hosmer-Lemeshow test showed good calibration in the training data set (*P* = 0.17) and the test set (*P* = 0.62), indicating high accuracy of the prediction model. Decision curves (Figure 6) were used to evaluate the clinical utility of the combined clinical-radiomics prediction model. DCA demonstrated that if the threshold probability was greater than 0.4 in clinical decision making, the nomogram is superior to the FLAIR radiomics and the clinical model. The sensitivity, specificity, accuracy, negative predictive value and positive predictive value of FLAIR radiomics, clinical features and clinical-FLAIR radiomics in predicting the prognosis of acute ischaemic stroke after thrombolytic therapy in the training set and the test set are shown in Table 5. ROC curves were used to evaluate the efficacy of the three models (clinical, radiomics, and clinical-radiomics models) in predicting the prognosis of AIS after thrombolysis in the training set and the test set. Among them, the clinical-radiomics model (radiomics nomogram) had the highest prediction efficacy, and the AUCs of the training set and the test set were 0.94 (95% CI: 0.90–0.98) and 0.94 (95% CI: 0.87–1), respectively, which was validated in the independent validation set1 (AUC = 0.95; 95% CI 0.88–1) and validation set 2 (AUC = 0.90; 95% CI 0.768–1) (Figure 7). DeLong test was used to compare the nomogram ROC curve with clinical model in both training set (*Z* = 2.4278, *P* = 0.01519) and test set (*Z* = 1.4023, *P* = 0.1608).

**TABLE 3:** The clinical model of multivariate regression results.

Variable	OR	CI.95	*P*-value
The overall CSVD burden score	1.96	(0.99; 3.86)	0.053
Admission NIHSS	1.37	(1.21; 1.56)	<1e^−04^
HT	2.87	(0.77; 10.72)	0.118

**TABLE 4:** The nomogram model of multivariate regression results.

Variable	OR	CI.95	*P*-value
The overall CSVD burden score	2.70	(1.11; 6.60)	0.029
Admission NIHSS	1.23	(1.05; 1.44)	0.008
HT	2.24	(0.45; 11.01)	0.322
Radscore	3.13	(1.73; 5.67)	0.000

**TABLE 5:** The prediction results of the three models in the training and test sets.

Model	Accuracy	Accuracy lower	Accuracy upper	Sensitivity	Specificity	Pos. pred. value	Neg. pred. value
FLAIR radiomics (Train)	0.822	0.741	0.886	0.933	0.753	0.700	0.948
FLAIR radiomics (Test)	0.792	0.650	0.895	0.833	0.767	0.682	0.885
Clinics (Train)	0.847	0.770	0.907	0.733	0.918	0.846	0.848
Clinics (Test)	0.854	0.722	0.939	0.722	0.933	0.867	0.848
Nomogram (Train)	0.839	0.760	0.900	0.889	0.808	0.741	0.922
Nomogram (Test)	0.854	0.722	0.939	0.789	0.897	0.833	0.867

The overall CSVD burden score, Admission NIHSS, HT and Radscore are vertically corresponding to the “points” in the first row. Finally, these “points” are added together to obtain the “total points” in the last row as the poor prognosis risk score, and the probability of poor prognosis of each stroke patient can be obtained.

Figures 5A–D shows the calibration curves of the three groups of models in the training set, test set and validation set 1 and validation set 2, reflecting the potential clinical net benefits of the models. The horizontal axis shows the nomogram predicting the prognosis of acute ischaemic stroke, and the vertical axis shows the actual prognosis of acute ischaemic stroke. The prediction efficacy is better if the solid line is closer to the grey line.

In Figure 6 decision curves show the decision curves of the three groups of models in the training set, reflecting the prediction efficiency of the models. The horizontal axis represents the threshold probability, the vertical axis represents the net benefit, and the solid lines represent the benefit curves of the models. DCA demonstrated that if the threshold probability was greater than 0.4 in clinical decision making, the nomogram is superior to the FLAIR radiomics and the clinical model.

Figures 7A–D shows the ROC curves of FLAIR radiomics, the clinical model, and the nomogram in the training set, test set, and validation set 1 and validation set 2, reflecting the comprehensive comparison of sensitivity and specificity among the three models. The AUC values of the test group were 0.89, 0.89, and 0.94, respectively.

## 4. Discussion

Stroke is a serious threat to human health worldwide. Early diagnosis and prognosis assessment are crucial for the management of AIS. The prognosis of acute ischaemic stroke is different due to differences in cerebrovascular reserve, collateral circulation and risk factors (Yang et al., 2013). DWI shows lesions within a short time (a few hours) after onset (Chalela et al., 2007), which represents the core of ischaemic stroke. Many studies have shown that the volume of stroke is correlated with clinical functional outcome (Jiang et al., 2020). In addition, the boundary between the core area and the penumbra is clear, which is easy to identify and delineate, and the consistency is good. FLAIR images have high resolution, long scanning time, and relatively more image information. Quan G et al. showed that radiomics features extracted from FLAIR and ADC can be used as biomarkers for predicting adverse clinical outcomes of AIS and can also improve the predictive performance when added to the combined model (Quan et al., 2021). The overall CSVD burden score is independently related to the severity of neurological deficits and the clinical outcome of AIS. Liu et al. (2019) showed that the overall CVSD burden score is a reliable predictor of poor prognosis of AIS after IV RT-PA treatment. In this study, ROIs were collected three times for each patient, and the correlation coefficient (ICC) was used to test the consistency of ROIs within and between observer groups, which ensured the reproducibility of extracted radiomics features. Radiomics has been used to evaluate the prognosis and treatment of ischaemic stroke (Wang et al., 2020). In this study, 201 patients with acute ischaemic stroke were enrolled to explore the value of radiomics based on DWI and FLAIR combined with the overall CSVD burden score in predicting the prognosis of ischaemic stroke and to create a method that can be used in the management strategy of ischaemic stroke. Our study showed that the AUC value of the FLAIR model in estimating clinical outcomes of acute ischaemic stroke after thrombolysis was 0.922, 95% CI: 0.876–0.968, which was higher than that of the DWI model (AUC = 0.875, 95% CI: 0.815–0.935) and DWI-FLAIR model (AUC = 0.895, 95% CI: 0.840–0.950). Tanriverdi et al. (2016) found that the increase in FLAIR hyperintensity in ischaemic tissue indicated a good prognosis of patients after intravenous thrombolytic therapy. Tang et al. (2020) showed that radiomics features could be used as prognostic biomarkers based on penumbral quantification and developed a radiomics nomogram to predict the prognosis of thrombolysis in patients with AIS, the AUC of the radiomics nomogram predicting favourable clinical outcome reached 0.886 (95% CI 0.809–0.963) on day 7 and 0.777 (95% CI 0.666–0.888) at 3 months. However, their model was constructed from the radiomic features extracted from PWI and DWI. In clinical practice, most patients with ischaemic stroke receive only routine test sequences. Wang et al. (2021) developed a clinical radiomics nomogram based on DWI, which showed good performance in predicting the prognosis of ischaemic stroke in the training cohort [AUC = 0.80; 95% confidence interval (CI) 0.75–0.86], which was validated in the validation cohort (AUC = 0.73; 95% CI 0.63–0.82). However, their study only included DWI sequences and studied all AIS patients, while we only collected patients who could receive thrombolytic therapy. Our model fitted well and the AUC value of FLAIR model was higher than that of DWI and DWI-FLAIR model, although there were no significant differences between the three models. We finally select FLAIR model to establish Normogram to solve the clinical problem, because radiomics is more dependent on image resolution and heterogeneity, and FLAIR sequence resolution shows more information than DWI sequence. However, the combination of DWI and the FLAIR radiomics model did not improve the evaluation performance, which may be caused by the mutual interference of the extracted radiomics features.

In this study, a total of 11 features related to the prognosis of ischaemic stroke after thrombolysis were screened by a FLAIR radiomics model, including 1 shape feature and 10 texture features. The small area low grey level emphasis (SALGLE), zone variance (ZV), large area high grey level emphasis (LAHGLE), large area emphasis (LAE), size zone non-uniformity (SZN), small dependence low grey level emphasis (SDLGLE) and so on are of great significance for the prognosis of patients with acute ischaemic stroke. SALGLE measures the proportion of small and dark areas of the image. LAHGLE measures the proportion of areas with brighter and larger dimensions. ZV is the change in the measured volume. LAE measures the distribution of large focal areas, and if the value is larger, the texture is coarser. SZN measures changes in the volume of the size region in the image. SDLGLE measures the strong correlation with the dispersion of the darker parts of the image. Among the 11 features, SALGLE and LAHGLE had the greatest relative weights. All of these features reflect the heterogeneity of infarcts and if the value is higher and the prognosis of stroke is poor.

The poor prognosis of stroke patients is related to many factors, such as age, blood pressure, previous neurological disorder, admission NIHSS score, collateral circulation, white matter hyperintensity, cerebral microbleeds and so on. This study showed that the overall CSVD burden score, HT, and admission NIHSS score were associated with ischaemic stroke prognosis. CSVD is a chronic disease of the whole brain, and the imaging findings are often more severe than the clinical manifestations. CSVD imaging markers include lacunes, WMHs, CMBs, and PVSs, which often coexist. Overall, these imaging markers may reflect an overall status of the distal small artery or arteriole bed, and a moderate-to-severe overall CSVD burden may represent an overall more vulnerable cerebral microcirculation (Arba et al., 2016). Therefore, the overall CSVD burden score may be more suitable for evaluating the overall effect of CSVD on the brain. The benefits and risks of intravenous thrombolysis in patients with CSVD should be individually evaluated to reduce the incidence of cerebral haemorrhage and poor prognosis after thrombolysis in ischaemic stroke. Tian et al. (2022) showed that the status of CSVD and infarction number predicted recurrent stroke in patients with acute minor stroke and TIA. Chen et al. (2020) believe that CSVD is associated with more disability and bleeding events, may represent different vascular lesions, and plays different roles in the outcome of stroke. Studies have shown that the overall CVSD score is a reliable predictor of adverse AIS outcome after IV RT-PA treatment, and CSVD is associated with endothelial dysfunction and blood−brain barrier leakage, which may lead to a larger stroke volume and a worse prognosis after intravenous thrombolytic therapy after stroke (Arba et al., 2019). As a common imaging marker of CSVD, WMHs can affect the prognosis of stroke through various mechanisms. In the hyperacute phase of ischaemic stroke, WMH may affect the infarct volume and is related to the increase in infarct area, thus affecting the prognosis (Helenius et al., 2017). The clinical outcome of AIS after thrombolysis is related not only to vascular recanalisation and collateral circulation but also to the increase in vascular bed resistance and the decrease in cerebral blood flow regulation function after cerebral small vessel disease. In the acute phase of IS, this regulatory dysfunction leads to further reduction of cerebral perfusion and collateral circulation dysfunction, resulting in the expansion of ischaemic penumbra and increased risk of poor prognosis. Lacunes account for approximately a quarter of the total number of ischaemic strokes. The overall CSVD burden score in this study can correct the limitations of individual imaging biomarkers, and the radiomics nomogram can predict the risk of poor outcome after RT-PA treatment, especially in patients with two or more severe CSVD imaging markers, and more accurately predict the outcome of intravenous thrombolysis in ischaemic stroke. Our study show the radiomics nomogram presented good discriminatory potential in the training set (AUC = 0.94; 95% CI: 0.90–0.98) and test set (AUC = 0.94; 95% CI: 0.87–1), which was validated in the validation set 1 (AUC = 0.95; 95% CI 0.88–1) and validation set 2 (AUC = 0.90; 95% CI 0.768–1). DeLong test was used to compare the nomogram ROC curve with clinical model in both training set (*Z* = 2.4278, *P* = 0.01519) and test set (Z = 1.4023, *P* = 0.1608), and the difference was not statistically significant in the test set. However, it demonstrated good calibration, and decision curve analysis confirmed the clinical value of this nomogram. DCA demonstrated that if the threshold probability was greater than 0.4 in clinical decision making, the nomogram is superior to the FLAIR radiomics and the clinical model.

There were also some limitations in this study. Firstly, the sample size is relatively small, which may lead to overfitting. So, our study also used patient data with different MRI scanners and other two clinical centres as two external validation sets, and the repeatability and consistency were good. Secondly, this study is a retrospective study, therefore our results obtained from the consecutive AIS patients to reduce the choice of selection bias. Finally, patients with lacunar infarction and posterior circulation stroke were excluded from our study. Lacunar infarction has a small lesion and a slight neurological defect, and the prognosis is generally good. Posterior circulation stroke accounts for about 20% of ischaemic stroke, and is generally more serious than anterior circulation stroke, with higher disability rate and mortality. In order to ensure strict grouping and no bias in the radiomics results, lacunar infarction and posterior circulation infarction were excluded from this experiment. At present, we are following up the patients with posterior circulation infarction, and we plan to further study them with radiomics.

In conclusion, this study shows that the FLAIR radiomics model have a similar performance with the DWI radiomics model and DWI-FLAIR radiomics model. Clinical combination with the FLAIR radiomics nomogram shows good performance in predicting the prognosis of ischaemic stroke after thrombolysis, this could help clinicians plan rehabilitation for stroke patients.

## Data availability statement

The raw data supporting the conclusions of this article will be made available by the authors, without undue reservation.

## Ethics statement

Written informed consent was obtained from the individual(s) for the publication of any potentially identifiable images or data included in this article.

## Author contributions

QX and LG contributed to the conception of the study. QX and SD performed the data analyses. QX wrote the manuscript. LG contributed to the revision of the article. YZ, XZ, and DK helped perform the analysis with constructive discussions. XY, LJ, ZL, and WY contributed to the article data from other two clinical centers as external validation set. All authors contributed to the article and approved the submitted version.

## Footnotes

1
www.itksnap.org
